# Biologic complications in short implant-assisted versus conventional partial dentures

**DOI:** 10.1038/s41432-025-01140-9

**Published:** 2025-04-24

**Authors:** Nidhi Parmar

**Affiliations:** MClinDent Prosthodontics Specialty Trainee, Eastman Dental Institute, London, UK

**Keywords:** Removable prosthodontics, Dental implants, Dental implants, Fixed prosthodontics, Peri-implantitis

## Abstract

**A Commentary on:**

**Benzaquen S I, Ardakani M T, Tsigarida A** **et al***.*

Biologic complications with removable partial dentures assisted by short implants: A 34-month pilot randomized controlled clinical trial. *J Prosthet Dent* 2025; 10.1016/j.prosdent.2025.01.026.

**Design:**

This single-centre, parallel-arm, pilot randomised controlled clinical trial (RCT) was conducted in accordance with CONSORT guidelines. The primary objective was to compare the incidence of biologic complications between conventional removable partial dentures (CRPDs) and short implant-assisted removable partial dentures (IARPDs) in patients with Kennedy Class I bilateral distal-extension edentulism. A secondary objective was to evaluate implant survival and peri-implant outcomes over a mean follow-up period of 34 months.

**Case selection:**

Thirty-three partially edentulous adult participants (aged 36–87 years) were recruited. Included participants had sufficient bone height to accommodate 6 mm implants without the need for bone augmentation. A strict exclusion criteria included current smoking, uncontrolled diabetes, pregnancy or lactation, and use of immunosuppressive or antiresorptive medications. Random allocation to receive either CRPDs (*n* = 19) or IARPDs supported by two short implants (*n* = 14) was conducted after initial CRPD fabrication.

**Data analysis:**

Both implant- and non-implant-related biologic complications were recorded at baseline and annual follow-up visits up to four years. Parameters included caries, gingival inflammation, abutment tooth loss, peri-implant mucositis, peri-implantitis, and marginal bone level (MBL) changes. Statistical analyses were performed using chi-square tests, Fisher’s exact test and paired and unpaired t-tests, with significance set at *p* = 0.05.

**Results:**

Non-implant biologic complications affected 44.7% of abutment teeth in the CRPD group and 21.4% in the IARPD group; however, this difference was not statistically significant (*p* > 0.05). The most common complications were gingival inflammation and caries. One abutment tooth was lost in the CRPD group versus none in the IARPD group. Peri-implant mucositis and peri-implantitis were observed in 42.9% and 10.7% of implants, respectively. Implant survival was 81.2%. Most MBL occurred prior to prosthetic loading, with minimal loss thereafter.

**Conclusions:**

Both CRPDs and IARPDs are viable treatment options for patients with Kennedy Class I edentulism, with no significant difference in the incidence of biologic complications between groups.

## GRADE Rating:



## Commentary

The use of IARPDs has emerged as a potential solution to the biomechanical and functional limitations of CRPDs, particularly in Kennedy Class I patients. By placing posterior implants in distal extension areas, IARPDs aim to convert a Class I configuration to a Class III support system, thereby reducing cantilever forces and improving load distribution^1^. This theoretical advantage, demonstrated in finite element analyses and in vitro models has led to growing clinical interest in the modality^2,3^. However, evidence regarding its biologic benefits, particularly in terms of complication rates and implant survival, remains limited and inconsistent.

This RCT provides timely insight into this question by comparing biologic complications between IARPDs using 6 mm short implants and CRPDs over a 34-month follow-up^4^. Contrary to the hypothesis that implant support would reduce biologic complications affecting abutment teeth, the study found no statistically significant difference between groups. Gingival inflammation and caries were the most frequent complications, observed in both cohorts, with a complication rate of 44.7% in the CRPD group and 21.4% in the IARPD group. These findings are in line with previous observational studies, which have shown that removable partial dentures, are associated with increased plaque accumulation and compromised periodontal conditions on abutment teeth^5–7^. Interestingly, the presence of clasps in the IARPD group did not increase the incidence of biologic complications, contradicting earlier reports suggesting that clasp removal in IARPDs improves periodontal outcomes^8^.

Implant-related outcomes in this study were less favourable than previously reported. The overall implant survival rate of 81.2% over 34 months is lower than expected based on systematic reviews of short implants, which report survival rates between 86.7% and 100%^9–11^. Peri-implant mucositis and peri-implantitis were identified in 42.9% and 10.7% of implants, respectively. These rates align with prevalence estimates from the 2017 World Workshop on Periodontal and Peri-Implant Diseases, which report mucositis in 19–65% and peri-implantitis in 1–47% of implants^12^. The majority of marginal bone loss occurred between implant placement and baseline (prosthetic loading), a pattern consistent with early remodelling responses observed in both short and standard-length implants^13^. Notably, marginal bone gain was observed in two implants between years three and four, a phenomenon reported in select studies as evidence of late-stage mineralisation, particularly in short implant cases with adequate loading control^14^.

Despite these insights, several methodological limitations constrain the external validity of the findings, resulting in a moderate GRADE rating for the overall quality of evidence. As a pilot trial, the small sample size (*n* = 33) limited statistical power and increased susceptibility to type II error. While randomisation was performed, the study did not clearly report on allocation concealment or blinding of assessors—factors critical for minimising detection and performance bias^15^. Attrition beyond year two significantly reduced the sample available for long-term analysis, particularly regarding marginal bone level changes and late complications.

The absence of patient-reported outcome measures (PROMs) is another key limitation. Previous work by the same authors reported no significant difference in patient satisfaction between CRPD and IARPD groups, suggesting that the perceived benefit of implant assistance may not translate to improved quality of life^16^. Furthermore, factors such as occlusal loading, opposing dentition, soft tissue biotype, and implant site (molar vs premolar) were not stratified or adjusted for, despite their known influence on both tooth and implant-related outcomes^17–19^.

Conversely, the study’s methodological rigour in other areas strengthens its internal validity, meeting most criteria outlined within the CASP checklist. Calibrated clinicians conducted all measurements, radiographic protocols were standardised, and biologic outcomes were assessed at both tooth and implant levels, allowing for granular interpretation. The study was conducted in accordance with CONSORT guidelines, and its structured reporting of biologic complications adds valuable data to a field with limited prospective comparative trials.

From a clinical standpoint, while the addition of short implants may improve prosthesis stability and reduce tissue loading, they do not appear to significantly mitigate biologic complications compared with conventional designs. Moreover, they introduce new risks, including peri-implant disease and surgical failure, which must be carefully weighed against their potential benefits. Patient selection remains paramount, particularly in assessing oral hygiene capacity, systemic risk factors, and long-term compliance with maintenance protocols.

In conclusion, this trial highlights the complexity of translating biomechanical theory into clinical success. It suggests that while IARPDs can be a viable option, they should not be presumed superior in preventing biologic complications. Further high-quality, adequately powered RCTs with integrated PROMs and long-term follow-up are needed to determine the true clinical and biological value of IARPDs, particularly in populations with systemic or anatomical constraints.

Practice point

- While a clinically viable treatment in cases with limited bone availability, clinicians should weigh the potential benefits of IARPDs against the risks of peri-implant disease and implant failure, underscoring the importance of ongoing maintenance and patient selection.
